# Chemical Recognition Mechanism of *Telenomus remus* Preference for *Spodoptera frugiperda* Eggs Based on Metabolomics with GC-MS

**DOI:** 10.3390/insects17030321

**Published:** 2026-03-16

**Authors:** Chunyan Yi, Wenjuan Yu, Mao Wang, Cuicui Zhang, Lei Wang, Tianqin Fan, Yang Yang, Song Chen, Yanping Wang

**Affiliations:** Key Laboratory of Integrated Pest Management of Southwest Crops, Institute of Plant Protection, Sichuan Academy of Agricultural Sciences, Chengdu 610066, China; yichunyan2022@126.com (C.Y.); wjyu0906@163.com (W.Y.); maode2046@163.com (M.W.); zhangcuicui2023@outlook.com (C.Z.); wanglei02017@outlook.com (L.W.); fantianqin@outlook.com (T.F.); 19138907637@163.com (Y.Y.); chensong523@126.com (S.C.)

**Keywords:** *Telenomus remus*, *Spodoptera frugiperda*, GC-MS, semiochemical, host selection

## Abstract

Although *Telenomus remus* is an important parasitoid of *Spodoptera frugiperda*, the chemical basis for its host selection behavior remains unclear. To elucidate the chemical basis of this behavior, this study combined behavioral ecology and chemical ecology methods to systematically investigate the host location and recognition behaviors of this wasp, as well as the semiochemicals that regulate these behaviors. In Y-tube olfactometer assays, *T. remus* exhibited a significantly stronger olfactory preference for eggs of *S. frugiperda* over those of *S. litura* (*p* < 0.05) or the non-host *Ostrinia furnacalis*. A total of 759 metabolites belonging to 11 categories were identified via metabolomics analysis, and principal component analysis (PCA) clearly distinguished between host eggs and non-host eggs. Analysis of differential metabolites revealed that the significantly upregulated metabolites in host eggs mainly included aldehydes, ketones and esters, followed by hydrocarbons, alcohols and amines. Subsequently, we screened and verified the effects of the significantly upregulated metabolites in host eggs compared with non-host eggs on the host-selection behavior of *T. remus*, including indole, 2-hexanol, and trans-1,2-dimethylcyclohexane, as well as 2-heptadecanone and n-nonadecane—two alkane compounds which are specifically upregulated on the surface of *S. frugiperda* eggs. Behavioral validation demonstrated that 2-hexanol exerted a significant repellent effect on *T. remus*, whereas trans-1,2-dimethylcyclohexane exhibited a significant attractive effect on the parasitoid wasp. Among the metabolites specifically upregulated in *S. frugiperda* eggs, 2-heptadecanone exhibited significant attractive activity at concentrations ranging from 0.1 to 1.0 mg/mL. This study is the first to report that the cycloalkane compound trans-1,2-dimethylcyclohexane acts as a potential broad-spectrum chemical marker for *T. remus* to recognize the eggs of host species belonging to the family Noctuidae, while 2-heptadecanone may further enhance its preference for the optimal host *S. frugiperda*. These findings provide novel candidate molecular targets for the development of behavioral regulators targeting egg parasitoids against *S. frugiperda*.

## Simple Summary

*Telenomus remus* is a natural enemy of *Spodoptera frugiperda*, a major agricultural pest, during its egg stage. To elucidate how it locates and selects host eggs, we investigated its behavioral responses to eggs of different insect species and analyzed the chemical profiles of these eggs. The study found that this wasp shows a stronger preference for the eggs of its natural host, *S. frugiperda*. Through comparative chemical analysis, we found that the level of specific aldehydes, ketones, esters and other substances was significantly upregulated in the host eggs. Further experiments identified several key compounds: trans-1,2-dimethylcyclohexane, a cycloalkane, exhibited an attractant effect on the wasps and may serve as a universal signal for its recognition of noctuid host eggs; 2-heptadecanone could specifically enhance its preference for the optimal host, the eggs of *S. frugiperda*. In contrast, 2-hexanol exhibited a repellent effect on the wasps. This study is the first to identify the key chemical compounds underlying the host selection behavior of *T. remus*, thereby providing potential molecular targets for the future development of novel products that attract this wasp to achieve greater control of *S. frugiperda*.

## 1. Introduction

The fall armyworm, *Spodoptera frugiperda* (Smith), is recognized as one of the most destructive agricultural pests globally. Since its introduction into China in 2019, this pest has rapidly spread across 26 provinces (autonomous regions and municipalities directly under the Central Government), posing a substantial threat to the production security of staple crops nationwide (e.g., maize and sugarcane) nationwide [1,2]. Its broad adaptability, strong migratory capacity, and rapid evolution of resistance to chemical insecticides have made traditional control strategies increasingly untenable [3].

A variety of strategies have been developed to manage *S. frugiperda*. In some countries in the Americas, the widespread cultivation of transgenic *Bt* insect-resistant crops (e.g., cotton and corn) has achieved significant outcomes in mitigating the damage caused by this pest [4]. In addition, integrated control measures centered on biological control, primarily including the use of predators and parasitoids [5,6,7], pathogenic microorganisms [8], and biogenic pesticides [9,10,11,12], play a crucial role. These methods, along with sex pheromones, can all be applied for the control of *S. frugiperda*. In its native range, *S. frugiperda* has a highly diverse community of parasitic natural enemies, primarily within the orders Hymenoptera and Diptera. Within Hymenoptera, egg parasitoids primarily include the families Trichogrammatidae (e.g., *Trichogramma* spp.) and Scelionidae, and larval parasitoids mainly comprise Braconidae and Ichneumonidae. In the order Diptera, significant parasitoids are dominated by the family Tachinidae, with representative species including *Archytas marmoratus* (Townsend) and *Lespesia archippivora* Riley [4]. However, research on the interaction mechanisms between natural enemies and their hosts in China remains limited. Thus, there is an urgent need to develop natural control strategies based on these indigenous natural enemies.

*Telenomus remus* Nixon (Hymenoptera: Scelionidae) is an effective egg parasitoid of lepidopteran pests [13]. It possesses advantages such as a short generation time (10 days at 25 °C) and a wide temperature adaptation range (15–31 °C) [14] and thus, it is considered a promising biological control agent [15]. Additionally, this parasitoid wasp can parasitize a variety of noctuid pests, including *Spodoptera litura* Fabricius and *Spodoptera exigua* (Hübner) [16]. *Spodoptera frugiperda* egg masses are stacked and covered with dense villi, but *Telenomus remus* can overcome this limitation hindering effective parasitism by *Trichogramma pretiosum* by removing these villi and maintaining a parasitism rate of over 90% even for the innermost eggs. Therefore, it shows high parasitism efficiency, identifying it as the most promising candidate natural enemy at present [17,18]. Although this wasp is polyphagous, its role as a dominant egg parasitoid of *S. frugiperda* suggests that specific chemical signals may exist between the pest and the natural enemy, mediating its precise host localization behavior–a mechanism that warrants further in-depth investigation. For egg parasitoid wasps, the volatiles from the host egg surface serve as critical information sources to conduct long-distance localization and short-distance recognition [19]. However, the key egg surface semiochemicals that regulate host selection by *T. remus*, particularly its preference for *S. frugiperda* eggs, remain unknown. Numerous studies have demonstrated that larval parasitoids rely on herbivore-induced plant volatiles (HIPVs), whereas egg parasitoids depend on oviposition-induced plant volatiles, indicating distinct strategies employed by larval and egg parasitoids in host recognition and location [20]. Volatile semiochemicals from host eggs serve as direct information sources or contact kairomones for many parasitoid wasps. As the egg stage represents an inactive period for the host, these volatile semiochemicals play a decisive role as critical information sources for parasitoid wasps to rapidly locate egg masses [21,22,23]. Although key semiochemicals provide more critical chemical cues for egg parasitoids to accurately recognize and locate hosts [17], research on the roles of host volatiles in host recognition and localization by egg parasitoids remains relatively scarce.

Egg and larval parasitoids differ significantly in their host recognition mechanisms. The key semiochemicals and their functional modes underlying the host recognition of *S. frugiperda* eggs by *T. remus* are unknown, hindering the targeted optimization of host selection in natural enemy rearing. This has resulted in a lack of molecular targets for designing behavioral regulators (e.g., attractants). We hypothesize that the preference of *T. remus* for *S. frugiperda* eggs is determined by specific volatile compounds (or specific proportional combinations) on the egg surface of *S. frugiperda*. To test this hypothesis, a behavioral phenotype–chemical phenotype correlation analysis strategy was employed. First, we quantified the preference gradient of *T. remus* for several host species (*S. frugiperda*, *S. litura*, *S. exigua*) and a non-host species (*Ostrinia furnacalis* Guenée) through rigorous behavioral assays. Subsequently, we employed GC-MS-based, widely targeted metabolomics to systematically dissect the variations in chemical profiles across different host eggs. Finally, we screened the key differential compounds and validated their biological activity in behavioral assays.

## 2. Materials and Methods

### 2.1. Materials

*Telenomus remus*, *Spodoptera frugiperda*, and *Ostrinia furnacalis* were maintained in our laboratory under controlled conditions: *Telenomus remus* at 26 ± 1 °C with a 16:8 h light:dark (L:D) photoperiod, while *S. frugiperda* and *O. furnacalis* were kept at 28 ± 1 °C with the same photoperiod. *Telenomus remus* was reared from *S. frugiperda* eggs, and the emerged adults were provided with a 10% (*v*/*v*) honey solution. *Spodoptera frugiperda* and *O. furnacalis* larvae were reared on an artificial diet prepared in our laboratory, and their adults were supplied with a 10% (*v*/*v*) honey solution. *Spodoptera litura* and *S. exigua* Pupae were purchased from Henan Jiyuan Baiyun Industrial Co., Ltd. (Keyun, China) and kept under the same conditions as *S. frugiperda* in our laboratory; they were then used after egg-laying.

The eggs for the odorant analysis experiment were divided into four groups—4 biological replicates each for Sf, Sl and Se, and 3 biological replicates for Of—for a total of 15 samples.

### 2.2. Experimental Methods

#### 2.2.1. Observation of Olfactory Behavioral Responses of *T. remus* to Different Treatments

The olfactory behavioral responses of *T. remus* females to the tested eggs were determined using a Y-tube olfactometer (Nanjing Xuelai Biotechnology Co., Ltd., Nanjing, China). Each arm of the Y-tube was 15 cm in length with an inner diameter of 1.6 cm, and the entire apparatus was kept in complete darkness throughout the experiment. The two arms of the Y-tube were connected to odor source bottles containing the treatment (e.g., *S. frugiperda* eggs) and the control. The airflow carrying the odor source was first filtered through an activated carbon filter and then humidified by a humidifier, and the air pump blowing air into the two arms was regulated by a flow meter, with a constant airflow rate of 200 m/min maintained for both arms. After connecting the Y-tube, pre-blowing was performed for 10 min to purge residual gases inside the apparatus. Using a fine brush, we placed a single female *T. remus* at the center of the main arm of the Y-tube. The behavior of *T. remus* behavior the tube was observed for 5 min, and a valid choice was recorded when the parasitic wasp crawled past the midpoint of either side arm.

The positions of the left and right arms of the Y-tube were swapped after every 5 tested *T. remus individuals*. A new Y-tube was replaced after every 10 tested *T. remus individuals*, with the positions of the sample and the control interchanged at the same time. Each parasitic wasp was used only once, with a minimum of 60 valid biological replicates. After each use, the Y-tube and odor source bottles were rinsed three times with 100% ethanol and then dried in an oven at 65 °C for 2 h; the connected rubber tubes were also dried in the oven at 65 °C for 2 h to eliminate residual odors. All experiments were conducted in a constant-temperature room maintained at 26 ± 1 °C under complete darkness, during the active period of *T. remus* (08:00–18:00).

#### 2.2.2. Determination of Egg Odor Metabolites by GC-MS

Sample extraction: In total, 0.2 g of the sample was transferred immediately to a 20 mL headspace vial (Agilent, Palo Alto, CA, USA). The vials were sealed using crimp-top caps with TFE-silicone headspace septa (Agilent). During SPME analysis, each vial was heated at 60 °C for 5 min; then, a SPME Arrow (Agilent) of 120 µm DVB/CWR/PDMS was exposed to the headspace of the sample for 15 min at 60 °C.

GC-MS conditions: After sampling, the VOCs from the SPME Arrow coating were desorbed in the injection port of the GC apparatus (Model 8890, Agilent) at 250 °C for 5 min. The identification and quantification of VOCs was carried out using an Agilent Model 8890 GC and a 7000D mass spectrometer (Agilent), equipped with a 30 m × 0.25 mm × 0.25 μm DB-5MS (5% phenyl-polymethylsiloxane) capillary column. Helium was used as the carrier gas at a linear velocity of 1.2 mL/min. The injector temperature was kept at 250 °C. The oven temperature was initially 40 °C (3.5 min), then increased by 10 °C/min to 100 °C, by 7 °C/min to 180 °C, at 25 °C/min to 280 °C, and held for 5 min. We recorded mass spectra in electron impact (EI) ionization mode at 70 eV. The quadrupole mass detector, ion source, and transfer line temperatures were set as 150, 230 and 280 °C, respectively. The MS was used in selected ion monitoring (SIM) mode to identify GC-MS conditions.

#### 2.2.3. Verification of the Behavior of Key Differential Compounds

Based on the results of metabolomics analysis, compounds with significant differences in the GC-MS test results were selected for behavioral response experiments. Using DMSO as an organic solvent additive, volatile standard samples with concentrations of 0.01, 0.1, 1, and 10 mg/mL were prepared with clean water. Then, 10 μL of the standard solution and an equal volume of control (solvent dilution with equivalent concentration) were pipetted onto filter paper of equal area (2 cm^2^) and placed in the two sample bottles of the Y-shaped olfactometer. The specific operational steps of the experiment are consistent with those in Section 2.2.1.

### 2.3. Data Processing

Statistical analyses were conducted on the data collected from the Y-tube olfactometer using SPSS version 26.0. The olfactory behavioral responses of *T. remus* to different treatments were analyzed using binary logistic regression within the framework of the Generalized Linear Model (GLM). For the behavioral validation experiments focusing on key differential compounds, the Chi-square test was used to determine the significance of the differences [24,25,26]. Gas chromatography–mass spectrometry (GC-MS) was used to determine the odor metabolites of eggs, and the raw data obtained from mass spectrometry analysis were processed using the MassHunter software version 10.0 for subsequent qualitative and quantitative analysis. The Personalbio Cloud Platform was adopted to perform principal component analysis (PCA), cluster heatmap analysis, and Venn diagram analysis of metabolic components. To identify differential metabolites among the groups, variables with values above the threshold of variable importance in the projection (VIP) from orthogonal partial least squares–discriminant analysis (OPLS-DA) were selected. For the convenience of observing variation trends in the relative contents of differential metabolites, a heatmap was plotted using the differentially expressed metabolites selected with the screening criteria.

## 3. Results

### 3.1. Olfactory Preference of T. remus for Four Types of Eggs

**Figure 1:**
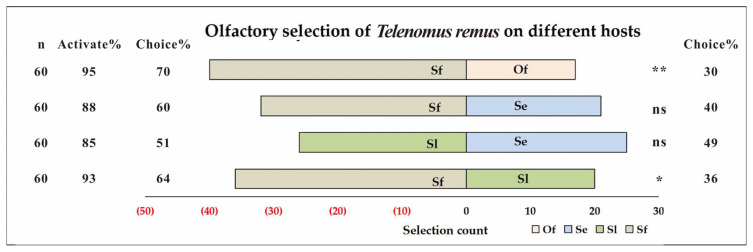
Olfactory selection of *Telenomus remus* on different hosts. Note: Sf = *Spodoptera frugiperda*; Of = *Ostrinia furnacalis*; Sl = *S. litura*; Se = *S. exigua*; Activate (%) = (Number of wasps that entered the arms/Total number of wasps tested) × 100; Choice (%) = (Number of wasps choosing a specific odor arm/Number of wasps that entered the arms) × 100. ns represents no significant difference at the 0.05 level; * indicates a significant difference at the 0.05 level; ** denotes significant difference at the 0.01 level.

The results of the Y-tube olfactometer bioassay (Figure 1) indicated that *T. remus* exhibited a distinct preference gradient towards different host eggs. When *S. frugiperda* and *S. litura* eggs were placed at the two ends of the Y-tube, the olfactory response of *T. remus* to *S. frugiperda* (selection rate = 64%) was significantly higher than to *S. litura* (selection rate = 36%) (*p* = 0.035). In the choice test between *S. frugiperda* (selection rate = 60%) and *S. exigua* (selection rate = 40%) eggs, there was a numerical difference in the olfactory preference of *T. remus*, but this was not statistically significant (*p* = 0.134). No difference was observed in the olfactory behavior of *T. remus* when presented with a choice between *S. litura* (selection rate = 51%) and *S. exigua* eggs (selection rate = 49%) (*p* = 0.889). However, when *S. frugiperda* (selection rate = 70%) and *O. furnacalis* (selection rate = 30%) eggs were the two options, *T. remus* showed an extremely significant olfactory preference for *S. frugiperda* eggs (*p* = 0.003). In summary, the host selection preference of *T. remus* can be ranked as Sf > Se ≈ Sl > Of.

### 3.2. Overview of Metabolite Profiles Based on Behavioral Preferences

**Figure 2:**
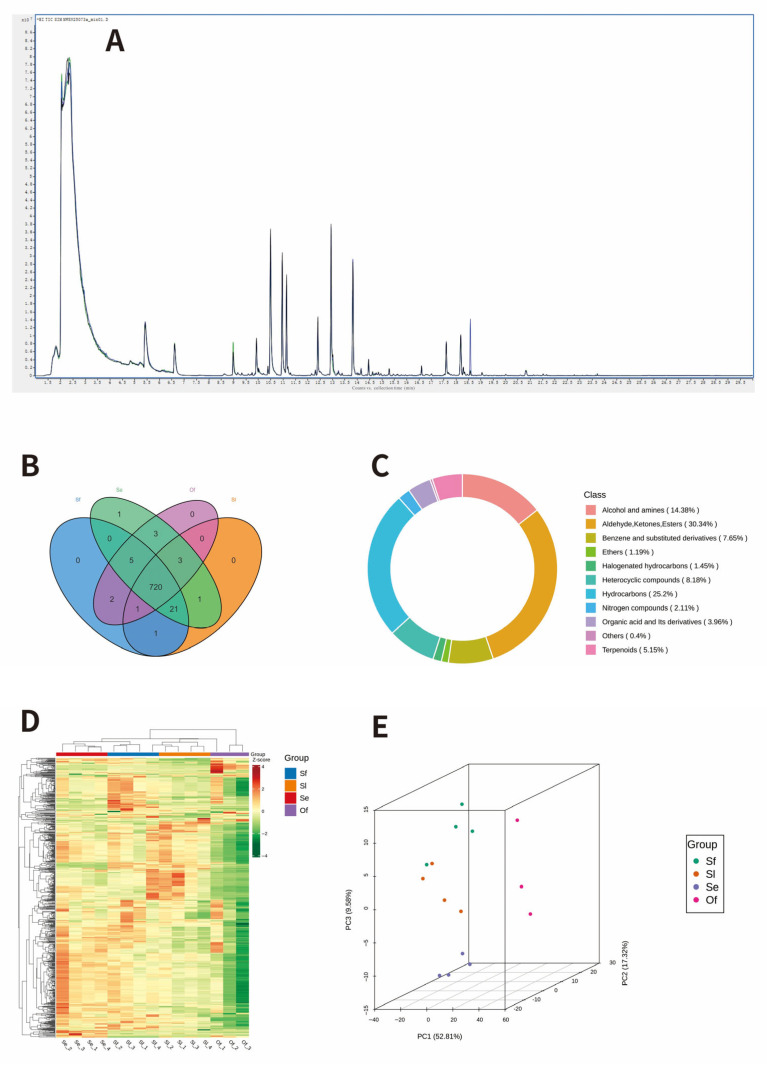
Overview of metabolite profiling. (**A**) QC sample mass spectrometry detection TIC overlap diagram; (**B**) Venn; (**C**) Metabolite category composition ring diagram; (**D**) The overall clustering heatmap of the sample; (**E**) Principal component analysis (PCA) score plot.

To elucidate the chemical basis underlying this behavioral preference, we performed a GC-MS widely targeted metabolomic analysis on the four species of eggs. The total ion current (TIC) chromatograms of the quality control (QC) samples are presented in Figure 2A. The overlapping of the ion peaks among the samples was excellent, with uniform peak shapes and stable distributions, indicating that the experimental process was stable and the data were reliable. A total of 758 metabolites belonging to 11 categories were detected. Among these, 750, 747, 754, and 734 metabolites were identified from the eggs of *S. frugiperda*, *S. litura*, *S. exigua*, and *O. furnacalis*, respectively (Table 1). The number of common metabolites shared by all four egg species was 720 (Figure 2B). In terms of the number of chemical categories, aldehydes, ketones and esters accounted for the highest proportion (30.34%), followed by hydrocarbons (25.20%), alcohols, and amines (14.38%) (Figure 2C).

Cluster heatmap analysis (Figure 2D) revealed that all samples could be divided into two distinct clusters: the eggs of the *T. remus* host species (Sf, Sl, and Se) were grouped into one cluster, with similar metabolite compositions and contents; whereas the eggs of *O. furnacalis* (Of), a non-host species of *T. remus*, formed a separate cluster on its own. This result indicated that the samples within each group exhibited good aggregation repeatability and low variation in dispersion. This finding was further confirmed by the principal component analysis (PCA) score plot (Figure 2E): the host and non-host eggs showed a clear spatial separation with significant inter-group differences. The first principal component (PC1) explained the largest proportion of the total variance, which distinctly differentiated the host from non-host identities.

### 3.3. Screening of Differential Metabolites

Based on the OPLS-DA model analysis, differential metabolites (DAMs) were screened using the following criteria: variable importance in projection (VIP) > 1, *p* < 0.05, and fold change (FC) > 1 or <1. These were defined as the thresholds for significantly upregulated and down-regulated differential metabolites. In the Se vs. Of comparison, 197 metabolites were identified as upregulated and 11 as downregulated. For Sf vs. Of, 180 metabolites were upregulated and 9 were downregulated. In the Sl vs. Of comparison, 190 metabolites were upregulated and 18 were downregulated (Table 2). Among these upregulated metabolites, the dominant chemical classes were aldehydes, ketones and esters, followed by hydrocarbons, alcohols and amines.

#### 3.3.1. Analysis of Common Differential Metabolites Between Host Eggs and Non-Host Eggs of *T. remus*

**Figure 3:**
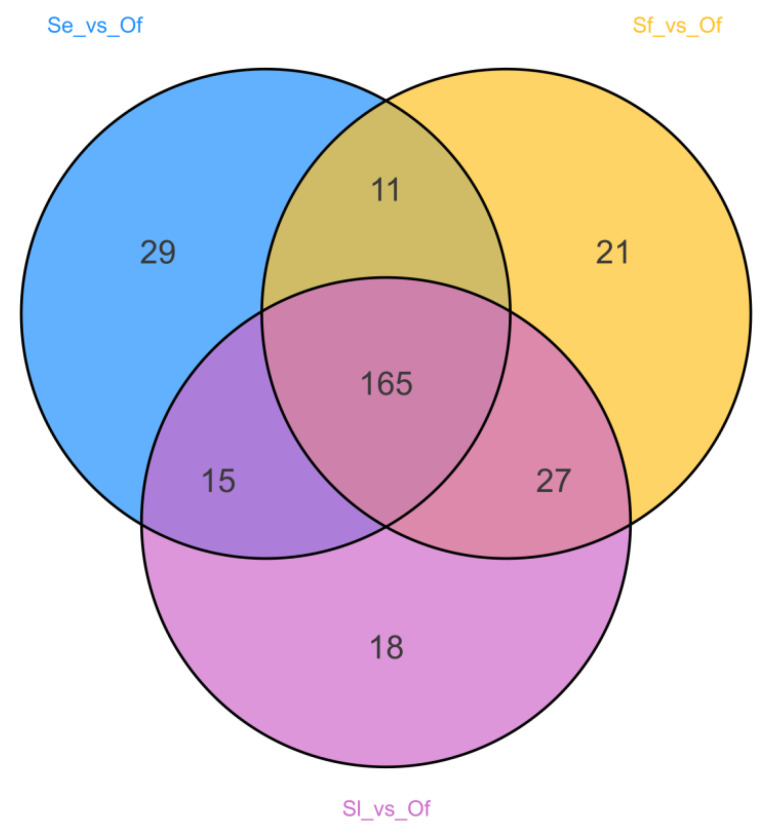
Overall Venn diagram comparing Se vs. Of, Sf vs. Of, Sl vs. Of.

**Figure 4:**
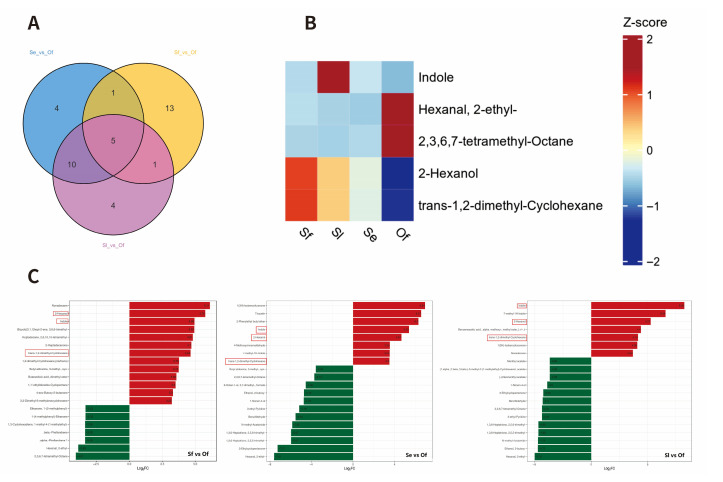
Differences in host species Sf, Sl and Se of *T. remus* compared with non-host species Of. (**A**) Venn diagram of top 20 fold-change differences in Se vs. Of, Sf vs. Of, Sl vs. Of; (**B**) Heatmap of co-upregulated and co-downregulated metabolites in Se vs. Of, Sf vs. Of, Sl vs. Of; (**C**) Bar chart of top 20 fold-change differences in Se vs. Of, Sf vs. Of, Sl vs. Of.

In the Venn diagram analysis (Figure 3), 165 common DAMs were identified across the three comparison groups, namely, *S. exigua* vs. *O. furnacalis*, *S. frugiperda* vs. *O. furnacalis*, and *S. litura* vs. *O. furnacalis*. A further analysis was conducted on the top 20 compounds ranked by fold change (FC value) among these common DAMs (Figure 4). Specifically, the *S. exigua* vs. *O. furnacalis* group contained 8 upregulated DAMs and 12 downregulated DAMs; the *S. frugiperda* vs. *O. furnacalis* group had 13 upregulated DAMs and 7 downregulated DAMs; and the *S. litura* vs. *O. furnacalis* group included 7 upregulated DAMs and 13 downregulated DAMs.

Across these three comparison groups, we identified three commonly upregulated DAMs, indole, 2-hexanol, and *trans*-1,2-dimethylcyclohexane, as well as two commonly downregulated DAMs: 2-ethylhexanal and 2,3,6,7-tetramethyloctane.

#### 3.3.2. Screening of Specific Differential Metabolites Related to Oviposition Preference of *S. frugiperda*

**Figure 5:**
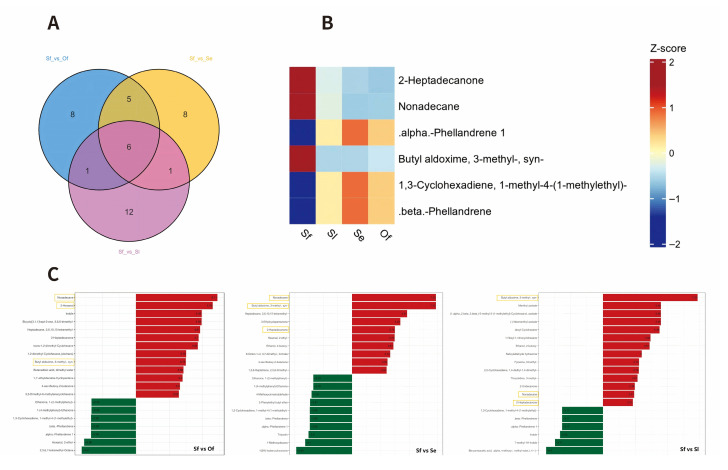
Comparison of Preference-Specific Signals Between Sf Eggs (Host of *T. remus*) and Other Host/Non-Host Eggs. (**A**) Venn Diagram of Top 20 Fold-Change Differences in Sf vs. Se, Sf vs. Sl and Sf vs. Of; (**B**) Heatmap of Co-Upregulated and Co-Downregulated Signals in Sf vs. Se, Sf vs. Sl and Sf vs. Of; (**C**) Bar Chart of Top 20 Fold-Change Differences in Sf vs. Se, Sf vs. Sl and Sf vs. Of.

To investigate why *T. remus* exhibits a stronger preference for *S. frugiperda* (Sf) among the three noctuid host species, we further analyzed the differential metabolites (DAMs) from the pairwise comparisons of Sf vs. Se, Sf vs. Sl, and Sf vs. Of (Figure 5). Among the top 20 DAMs ranked by FC values, three were commonly upregulated—nonadecane, (syn)-3-methylbutyraldoxime, and 2-heptadecanone—and three were commonly downregulated—α-phellandrene-1, 1-methyl-4-(1-methylethyl)-1,3-cyclohexadiene, and β-phellandrene. All six metabolites belong to the terpenoid class of compounds (Figure 5).

### 3.4. Olfactory Behavioral Responses of T. remus to Differential Metabolites on the Egg Surface of S. frugiperda

**Figure 6:**
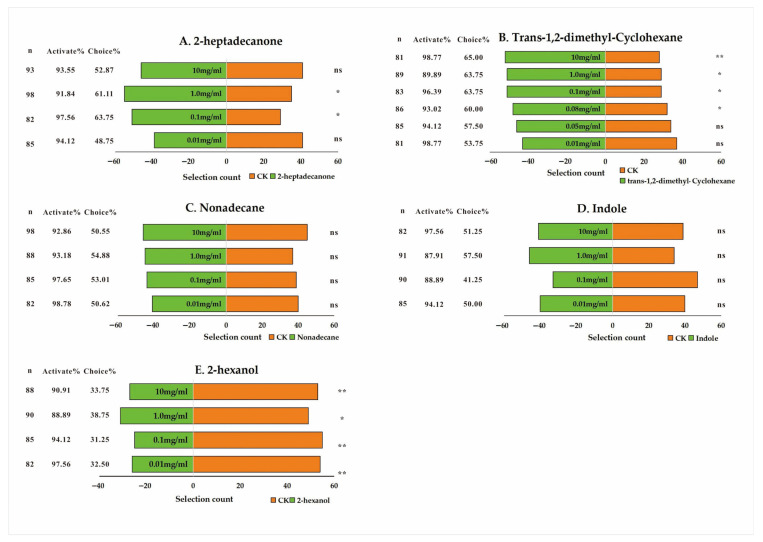
Olfactory responses of *T. remus* to differential metabolites. Note: ns represents no significant difference at the 0.05 level; * indicates a significant difference at the 0.05 level; ** denotes an extremely significant difference at the 0.01 level.

To verify the roles of the above-screened key differential metabolites in the host selection of *T. remus*, we conducted behavioral assays using a Y-tube olfactometer. First, three compounds (indole, 2-hexanol, and trans-1,2-dimethylcyclohexane) that were co-upregulated in host eggs were tested (Figure 6). The results showed that 2-hexanol exerted a significant repellent effect on *T. remus* at concentrations ranging from 0.01 to 10 mg/mL (*p* < 0.05) (Figure 6E). Trans-1,2-dimethylcyclohexane exhibited a significant attractive effect on *T. remus* at concentrations of 0.1 to 10 mg/mL (*p* < 0.05) (Figure 6B). Indole did not elicit any significant behavioral responses in *T. remus* at any tested concentrations (*p* > 0.05) (Figure 6D).

Second, two compounds specifically upregulated in *S. frugiperda* eggs were tested (nonadecane and 2-heptadecanone). The results showed that nonadecane did not exhibit significant behavioral effects at concentrations ranging from 0.01 to 10.0 mg/mL (Figure 6C). 2-heptadecanone exerted a significant attractive effect on *T. remus* at concentrations of 0.1 mg/mL and 1.0 mg/mL (*p* < 0.05), whereas no significant effects were observed at concentrations of 0.01 mg/mL or 10.0 mg/mL (Figure 6A).

## 4. Discussion

For the first time, this study quantitatively assessed, we quantitatively assessed the hierarchical oviposition preference of *T. remus* among several noctuid host eggs, which followed the order *S. frugiperda* > *S. exigua* ≈ *S. litura* > *O. furnacalis*. Furthermore, it was demonstrated that this preference gradient is significantly correlated with differences in the volatile chemical profiles present on the surfaces of the host eggs. Metabolomic analyses revealed that the metabolite profiles of host and non-host eggs were significantly separated in the PCA space, indicating that “host identity” acts as the primary factor shaping the surface chemical characteristics of eggs. This finding is consistent with previous research conclusions that parasitoid wasps rely on host chemical signals for host location [27,28]. The generally upregulated aldehydes, ketones, esters, and hydrocarbons in host eggs may constitute the fundamental chemical background for *T. remus* to recognize potential suitable hosts.

The use of semiochemicals for habitat and host localization constitutes a universal and crucial strategy adopted by insects. Notably, volatile compounds emitted from the body surfaces and eggs of phytophagous insects serve as key cues for host location by natural enemy insects. Previous studies have shown that this parasitoid wasp exhibits differential parasitism rates across various host species (e.g., *S. litura* and *S. exigua*), with a distinct active preference for *S. litura* eggs [29,30,31]. However, the behavioral assays conducted in this study confirmed that *T. remus* exhibits a distinct preference gradient toward different host eggs, with a significantly higher olfactory preference for *S. frugiperda* eggs than for *S. litura* eggs and non-host *O. furnacalis* eggs. Metabolite profiles differed significantly between host and non-host eggs, indicating that host identity acts as the primary factor influencing volatile compound composition. Screening of differential metabolites revealed that *T. remus* exhibited a significant preference for a novel class of bioactive cycloalkanes from *S. frugiperda* eggs, specifically, trans-1,2-dimethylcyclohexane. This behavior confirms that the wasps’ preference is directly associated with the specific chemical characteristics of volatile substances on the surface of its host eggs.

Studies have demonstrated that egg surface compounds exert a repellent effect on female parasitoid wasps, primarily because these compounds contain oviposition-marking components [32]. In contrast, the same compounds can attract corresponding natural enemy insects and trigger their orientation behavior [27,28,33]. In this study, trans-1,2-dimethylcyclohexane was identified for the first time as a commonly upregulated metabolite in host eggs of *T. remus* in comparison with non-host eggs. This compound was consistently upregulated in eggs of three Noctuidae host species, whereas it was detected at low levels in non-host eggs of the European corn borer. This characteristic enables it to potentially serve as a “host suitability marker” or “host-specific signal,” facilitating the rapid discrimination of Noctuidae (suitable hosts) from non-Noctuidae eggs (e.g., the European corn borer, unsuitable hosts) by *T. remus*. Compared with attractants frequently reported in the literature (e.g., n-alkanes and terpenoids), cycloalkanes have rarely been documented as parasitoid attractants, which provides novel insights into the chemical ecology of natural enemy insects.

Notably, indole—one of the metabolites commonly upregulated in the host eggs compared with the non-host *O. furnacalis* eggs of *T. remus*—exerted no significant effect on parasitoid wasps, whereas 2-hexanol elicits a distinct repellent effect on this species. To date, few studies have directly targeted 2-hexanol and egg parasitoids. 2-hexanol plays distinctly different ecological roles in different species, exhibiting high interspecific specificity; in particular, it has a significant repellent effect on females of *Cotesia ruficrus* (Haliday) [34]. Although it also serves as a semiochemical for habitat and host location in *Microplitis mediator* (Haliday) [35], this compound acts as a sex pheromone for the beetle *Glenea cantor* Fabricius [36]. Therefore, 2-hexanol may possess multiple ecological functions in insect–parasitoid interactions, with its specific effects depending on the compound concentration, target organisms, and ecological context.

As a nitrogen-containing aromatic heterocyclic compound, indole acts as a key signal triggering defense responses in plants such as maize and tea [37,38,39,40,41,42]. Indole exerts a significant attractive effect on *M. pallidipes* (Szepligeti), with the attractiveness intensifying as the concentration increases; however, it exhibits a repellent response at concentrations below 0.001 μg/mL [37]. In addition, indole has been extensively investigated in studies addressing whether herbivore-induced plant volatiles mediate tritrophic interactions by modulating the host attractiveness of herbivores [41,43]. In comparative assays of three host egg species (Se, Sf, and Sl) for *T. remus*, the parasitoid wasps exhibited a distinct oviposition preference for Sf eggs. The alkane compounds specifically upregulated on the surface of *S. frugiperda* eggs—including 2-heptadecanone and n-nonadecane—have been documented to mediate well-defined ecological functions across diverse insect systems. For instance, 2-heptadecanone has been identified as a defensive pheromone in *Uloma tenebrionoides* (White) and *Dolichovespula maculata* (Linnaeus) [44], whereas in *Campsomeris chlorideae* (Uchida), it acts as a key component of female sex pheromones, with its primary role being the specific attraction of conspecific males and the subsequent initiation of courtship and mating behaviors [45]. One study found that n-nonadecane, a cuticular hydrocarbon from the stink bug host *Nezara viridula* (L.), is a key chemical signal for the egg parasitoid *Trissolcus basalis* (Wollaston) to discriminate between female and male hosts [46]. The results of our study confirmed that nonadecane, a Sf-specific upregulated differential metabolite, exerted no significant effect on *T. remus*, whereas 2-heptadecanone at concentrations of 0.1 mg/mL and 1.0 mg/mL elicited a distinct attractive effect on the parasitoid wasps. The specific upregulation of these compounds on the surface of *S. frugiperda* eggs suggests that they are unlikely to act as general attractants; instead, they may serve as key signals associated with the identity of the specific host (*S. frugiperda*). Their unique combination or concentration might constitute a “chemical fingerprint” for Sf eggs, thereby guiding the directional host selection behavior of *T. remus*. In the context of egg surface chemical communication, 2-heptadecanone may enhance host identity recognition and guide parasitoid wasps to make optimal foraging choices. This compound and trans-1,2-dimethylcyclohexane may form a signal hierarchy: the latter provides broad-spectrum information indicating “a Noctuidae egg,” whereas the former further transmits specific signals specifying “a *S. frugiperda* egg.”

In summary, the host egg recognition and localization behaviors of *T. remus* rely on the specific composition of semiochemicals on the egg surface. Our findings are both consistent with and distinct from those of numerous previous studies on the regulation of host localization behavior, a discrepancy that may be due to long-term selective evolution. Co-upregulated compounds such as nonadecane and indole elicited no significant behavioral responses in *T. remus*, further indicating that the efficacy of individual compounds is relatively limited in complex chemical contexts. Instead, parasitoid host localization often depends on the recognition of specific compound blends or holistic chemical fingerprints. Cycloalkanes or specific terpenoids have been identified as species-specific attractants for parasitoids in other insect systems, which supports the potential core role of the specific compounds identified in this study in driving parasitoid host preference. These results demonstrate that the same compound may exert drastically different functions across distinct species or ecological contexts; namely, specific semiochemicals on host eggs may be ineffective for certain parasitoid species but could attract other taxa (e.g., predatory insects), with their ecological functions being determined by the ecological roles and evolutionary histories of the recipient organisms [47].

By integrating behavioral and chemical ecology approaches, this study systematically elucidated the chemical basis underlying host selection preference in *T. remus* and identified novel compounds with behavioral regulatory activities, particularly trans-1,2-dimethylcyclohexane. These findings hold substantial theoretical and practical significance. Future research should focus on three key aspects: (1) validating the practical attractive efficacy of these pivotal compounds under field conditions; (2) exploring the synergistic or antagonistic effects of these compounds when combined at different ratios; and (3) employing electrophysiological techniques (e.g., electroantennography, EAG) and molecular biological approaches to dissect the interaction mechanisms between the olfactory receptors of *T. remus* and these key compounds, thereby deciphering the recognition code at the molecular level.

## 5. Conclusions

This study clarified the significant behavioral preference of *T. remus* for *S. frugiperda* eggs and, through GC-MS metabolomics combined with behavioral validation, revealed that trans-1,2-dimethylcyclohexane may serve as a potential broad-spectrum chemical marker for *T. remus* to recognize host eggs of the Noctuidae family. Conversely, 2-heptadecanone—a metabolite specifically upregulated in *S. frugiperda* eggs—may play a potentiating role in mediating the preference for optimal hosts. Additionally, 2-hexanol—a metabolite commonly present in host eggs—may act as a defensive component that exerts a repellent effect on parasitoid wasps. These findings not only advance our understanding of the chemical mechanisms underlying parasitoid host selection, but also provide critical scientific evidence and novel molecular targets for developing new technology for the management of *S. frugiperda* based on semiochemicals.

## Figures and Tables

**Table 1:** Components of volatile metabolites from different eggs.

Class I	Sf	Sl	Se	Of	QC
Aldehyde, Ketones, Esters	226	227	230	222	230
Hydrocarbons	191	190	190	184	191
Alcohol and amines	109	108	108	107	109
Heterocyclic compounds	62	60	62	61	62
Benzene and substituted derivatives	58	57	58	56	58
Terpenoids	36	39	38	37	39
Organic acid and its derivatives	30	30	30	28	30
Nitrogen compounds	16	14	15	16	16
Halogenated hydrocarbons	11	11	11	11	11
Ethers	8	8	9	9	9
Others	3	3	3	3	3
Total	750	747	754	734	758

**Table 2:** Information on differential metabolites in different eggs.

Class	Se vs. Of		Sf vs. Of		Sl vs. Of		Sf vs. Se		Sf vs. Sl		Sl vs. Se	
	Up	Down	Up	Down	Up	Down	Up	Down	Up	Down	Up	Down
Aldehyde, Ketones, Esters	70	3	58	2	71	5	13	6	11	2	9	9
Hydrocarbons	49	4	37	2	40	4	6	0	6	0	6	3
Alcohol and amines	34	3	28	1	28	4	6	2	4	0	3	1
Heterocyclic compounds	13	1	17	1	15	2	3	0	3	2	4	2
Benzene and substituted derivatives	5	0	7	0	7	1	1	1	3	0	1	2
Terpenoids	5	0	10	3	8	0	2	3	5	3	2	0
Organic acid and its derivatives	11	0	11	0	11	0	0	0	0	0	0	0
Nitrogen compounds	5	0	8	0	5	2	1	0	4	0	0	3
Halogenated hydrocarbons	2	0	2	0	3	0	1	0	0	0	1	0
Ethers	3	0	2	0	2	0	0	1	0	0	0	1
total	197	11	180	9	190	18	33	13	36	7	26	21

Note: In the Se vs. Of group: Up represents DAMs that are upregulated in Se relative to Of, and Down represents DAMs that are downregulated in Se relative to Of.

## Data Availability

The original contributions presented in this study are included in the article. Further inquiries can be directed to the corresponding author.
